# High-dose 8% capsaicin patch in treatment of chemotherapy-induced peripheral neuropathy: single-center experience

**DOI:** 10.1007/s12032-017-1015-1

**Published:** 2017-08-17

**Authors:** Iwona Filipczak-Bryniarska, Roger M. Krzyzewski, Jakub Kucharz, Anna Michalowska-Kaczmarczyk, Justyna Kleja, Jarosław Woron, Katarzyna Strzepek, Lucyna Kazior, Jerzy Wordliczek, Tomasz Grodzicki, Krzysztof Krzemieniecki

**Affiliations:** 1 Department of Palliative and Pain Medicine, Jagiellonian University Medical College, Kraków, Poland; 2 Department of Neurosurgery and Neurotraumatology, Jagiellonian University Medical College, Kraków, Poland; 3 Department of Uro-Oncology, Maria Sklodowska-Curie Memorial Cancer Center, Institute of Oncology, Warszawa, Poland; 4 Department of Experimental and Clinical Surgery, Jagiellonian University Medical College, Michalowskiego 12 St, 31-126 Kraków, Poland; 5 Department of Oncology, University Hospital in Kraków, Kraków, Poland; 6 Department of Pharmacology, Jagiellonian University Medical College, Kraków, Poland; 7 Department of Interdisciplinary Intensive Care, Jagiellonian University Medical College, Kraków, Poland; 8 Department of Internal Medicine and Gerontology, Jagiellonian University Medical College, Kraków, Poland; 9 Department of Oncology, Jagiellonian University Medical College, Kraków, Poland

**Keywords:** Chemotherapy-induced peripheral neuropathy, Capsaicin, Pain, Neurotoxicity, Oxaliplatin

## Abstract

High-dose capsaicin patch is effective in treatment of neuropathic pain in HIV-associated neuropathy and diabetic neuropathy. There are no studies assessing effectiveness of high-dose capsaicin patch in treatment of chemotherapy-induced peripheral neuropathy. We sought to determine the effectiveness of treatment of pain associated with chemotherapy-induced peripheral neuropathy with high-dose capsaicin patch. Our study group consisted of 18 patients with clinically confirmed oxaliplatin-induced neuropathy. Baseline characteristic including underling disease, received cumulative dose of neurotoxic agent, neuropathic symptoms, prior treatment and initial pain level were recorded. Pain was evaluated with Numeric Rating Scale prior to treatment with high-dose capsaicin and after 1.8 day and after 8 and 12 weeks after introducing treatment. Patients were divided into two groups accordingly to the amount of neurotoxic agent that caused neuropathy (high sensitivity and low sensitivity group). Most frequent symptoms of chemotherapy-induced neuropathy were: pain (88.89%), paresthesis (100%), sock and gloves sensation (100%) and hypoesthesis (100%). Initial pain level was 7.45 ± 1.14. Mean cumulative dose of oxaliplatin after which patients developed symptoms was 648.07 mg/m^2^. Mean pain level after 12 weeks of treatment was 0.20 ± 0.41. When examined according to high and low sensitivity to neurotoxic agent patients with low sensitivity had higher pain reduction, especially after 8 days after introducing treatment (69.55 ± 12.09 vs. 49.40 ± 20.34%; *p* = 0.02) and after 12 weeks (96.96 ± 5.56 vs. 83.93 ± 18.59%; *p* = 0.04). High-dose capsaicin patch is an effective treatment for pain associated with chemotherapy-induced neuropathy in patients treated with oxaliplatin. Patients with lower sensitivity to neurotoxic agents have better response to treatment and pain reduction.

## Introduction

Chemotherapy-induced peripheral neuropathy (CIPN) is frequent complication of anti-neoplastic treatment with established frequency of 30–40% of patients undergoing chemotherapy [1]. It significantly reduces quality of life and may be the cause of dose limitation or even cessation of treatment [2]. CIPN can be induced by many chemotherapeutics such as: taxanes, vinca alkaloids, epothilone, bortezomib, thalidomide and especially platinum agents [3]. Oxaliplatin is the most frequent agent causing CIPN causing symptoms even after 2 years after cessation of treatment [4]. Currently, there are many emerging concepts for treatment of CIPN [5]. Treatment options that have proven effectiveness are duloxetine [6], venlafaxine [7] and gabapentinoids [8]. Opioids show very limited effects [9]. Promising concepts with very limited data consider topical menthol [10]. Fallon et al. [10] recently described that topical menthol improves pain, walking ability, mood and sensation in patients with CIPN. Other concept studied by Gewandter et al. [11] considered topical amitriptyline with ketamine, but received poor results in comparison with placebo.

Currently, high-dose topical capsaicin patch receives extensive attention. Capsaicin is an agonist of the transient receptor potential vanilloid receptor (TRPV1). It inhibits neural transmission in sensatory axons. Topical capsaicin patch has been proven effective for post-herpetic neuralgia [12, 13] and human immunodeficiency virus (HIV)-associated neuropathy [14]. On the other hand, large Cochrane Database review suggests that it should be used only as a last resort [15]. Despite growing interest in topical high-dose capsaicin patch in treatment of neuropathic pain, there are no studies describing effectiveness of capsaicin on CIPN [5]. The aim of our study is to determine the effectiveness of high-dose capsaicin patch in treatment of CIPN-associated pain.

## Methods

The study was conducted in accordance with the ethical principles of the Declaration of Helsinki, International Conference on Harmonization of Technical Requirements for Registration of Pharmaceuticals of Human Use (ICH) Good Clinical Practice, current US FDA guidelines, and local ethical and legal requirements. The study was approved by the Institutional Review Boards of all participating institutions. We received permission to conduct this study from the Jagiellonian University Bioethics Committee (KBET/235/B/2014). All patients provided written informed consent.

For the study, we qualified patients in stable state undergoing oxaliplatin-based chemotherapy for colon cancer who developed painful peripheral polyneuropathy, based on clinical history and neurological examination. The patients were asked to give their written consent before application of high-dose 8% capsaicin patch.

Between January 2013 and February 2014, twenty-one patients with chronic pain resulting from CIPN were referred by medical oncologists to application of a high-dose 8% capsaicin patch. All the patients were assessed and qualified by a specialist in neurology and palliative medicine. A questionnaire of neuropathic symptoms was used and included questions about the presence of numbness, tingling, pain or impaired sensory function in hands and/or feet, clumsiness in fingers, peripheral muscular weakness or difficulties in walking, and the patients replied with Yes or No answers. Symptoms and signs of neuropathic pain were evaluated including a history of sensory sensations such as tingling, numbness, sharp, burning, shooting, or electric-like pain, paresthesia, allodynia, hyperalgesia and hyperpathia and motor dysfunction (foot or wrist drop, symmetric motor weakness, difficulty buttoning a shirt or holding a pen), myalgias or muscle cramps. Neurological examination was performed by a neurologist. The neurological examination included motor assessment (muscle strength in the limbs), sensory testing (examination of touch, pinprick, cold, warmth, vibration and position sense and testing of allodynia for dynamic and static mechanical stimuli), testing of balance, coordination, and deep tendon reflexes. All patients met the inclusion criteria: age 18–72 years, diagnosed chemotherapy-induced peripheral sensory neuropathy, average pain intensity equal to or higher than 4 at screening for at least four consecutive days according to NRS, undamaged and non-irritated, dry skin at the sites of pain location, informed, written consent to participate in a clinical study. Patients were excluded when one or more of the following criteria were met: hypersensitivity to capsaicin, pregnancy, diabetes mellitus, patients with unstable or poorly controlled hypertension or a history of recent cardiovascular events (6 months) before starting treatment, patients using high doses of opioids in the treatment, patients in whom a high degree of opioid tolerance is suspected, patients in whom we observe lack of response to opioid analgesics administered orally in acute pain during the treatment procedure.

The patch application process took place in a dedicated minor operation theater. The patients selected for the treatment had their dynamic pain score recorded prior to application.

The patients were given a patient information leaflet prior to admission, and the procedure was explained and any concerns or questions answered. Capsaicin 8% patch was applied by a nurse and closely supervised by a physician. Skin on the application site could not be irritated, dry or intact, a topical anesthetic cream (EMLA) was applied 1 h before Qutenza patch application. The patch, which may be cut to shape, was used within 2 h of opening the foil pouch. Patients may require systemic analgesic medication or topical cooling at the site during and after treatment. They were encouraged to wear it for as long as possible, but it could be removed if the pain became intolerable. None of the adverse events occurred.

### Pain evaluation

Efficacy was measured using an 11-point Numerical Rating Scale (NRS) with 10 representing the worst pain imaginable and 0 representing no pain. Initial pain level was taken before administrating capsaicin patch and after 1 and 8 days after administration and after 8 and 12 weeks after administration of the patch.

### Statistical method

Elements of descriptive statistics were used (mean, standard deviation, percentage distribution). We used χ^2^ Pearson’s test to compare proportions. *T* test and Mann–Whitney U test were used as appropriate to compare continuous variables. All statistical calculations were preformed using STATISTICA 10.0 v. (Statsoft, Poland) software for Microsoft Windows.

## Results

Average pain score was 4 or above during the screening period, over a minimum of at least four consecutive days (using the NRS scale).

### Baseline characteristics

Eighteen patients were included into the study. All the patients received treatment according to the scheme and were involved in the evaluation of efficacy and safety. Among patients enrolled in the study, there were 12 women and 6 men, with a mean age of 62 years. Mean cumulative neurotoxic dose was 832.9 mg/m^2^; however, the beginning dose after which the symptoms of polyneuropathy appeared was 648.07 mg/m^2^. Average initial pain intensity was assessed at 7.67 ± 0.51 points in the group.

Because of the symptoms of chemotherapy-induced neuropathy in the period preceding the application of the patch 9 people did not receive any treatment for neuropathic pain. The treatment received by others is presented in Table 1. Eighteen patients out of twenty-one finished the complete observation. Three of the patients discontinued their participation in the study for personal reasons.

**Table 1:** Characteristic of treated patients

Characteristics	Value
Number of patients, *n* (%)	18 (100)
Age, years (SD)	62.67 (8.25)
Female, *n* (%)	12 (66.67)
*Cancer location*	
Colon, *n* (%)	18 (100)
Cumulative neurotoxic dose, mg/m^2^ (SD)	832.9 (111.53)
Beginning of symptoms after, mg/m^2^ (SD)	648.07 (331.12)
*Drugs prior to capsaicin*	
No treatment, *n* (%)	9 (50)
Gabapentin, *n* (%)	5 (27.78)
Pregabalin, *n* (%)	2 (11.11)
Amitriptyline, *n* (%)	2 (11.11)
SSRIs, *n* (%)	2 (11.11)
Transdermal lidocaine, *n* (%)	2 (11.11)
*Coexisting morbidities*	
Hypertension, *n* (%)	8 (44.44)
Ischemic heart disease, *n* (%)	5 (27.78)
*Symptoms*	
Pain, *n* (%)	16 (88.89)
Paresthesis, *n* (%)	18 (100.00)
Weakness, *n* (%)	15 (83.33)
Disequilibrium, *n* (%)	6 (33.33)
Dizziness, *n* (%)	2 (11.11)
Cramps, *n* (%)	3 (16.67)
Sock and gloves sensation, *n* (%)	18 (100.00)
Hypoesthesis, *n* (%)	18 (100.00)
Bathyanaesthesis, *n* (%)	17 (94.44)
Decreased deep tendon reflex, *n* (%)	18 (100.00)
Sensory ataxia, *n* (%)	6 (33.33)
Initial pain level	7.45 ± 1.14
Pain after 1 day, NRS, ±SD	4.95 ± 1.39
Pain after 8 days, NRS, ±SD	2.55 ± 1.14
Pain after 8 weeks, NRS, ± SD	1.20 ± 1.10
Pain after 12 weeks, NRS, ± SD	0.20 ± 0.41
WHO neuropathic score before treatment, ±SD	2.00 ± 0.65
ECOG neuropathic score before treatment, ±SD	2.35 ± 0.67
WHO neuropathic score after 12 weeks, ±SD	0.55 ± 0.51
ECOG neuropathic score after 12 weeks, ±SD	0.65 ± 0.48

### Efficacy

In the 12-week study, pain reduction in patients whose initial symptoms occurred after the administration of 648.07 mg/m^2^ of oxaliplatin was, respectively, 28% after the first day and 84% at 12 weeks after application of the patch. In the second group of patients in whom the symptoms appeared after the treatment with higher cumulative doses of oxaliplatin, reduction of pain was, respectively, 33% after the first day and 97% after 12 weeks. Statistical analysis of the results of the research shows a statistical significance between the treated groups of patients, which show as considerably higher levels of pain reduction after 8 days and 12 weeks for patients with the used cumulative doses (average of 20% after 8 days and about 14% after 12 weeks). The main measure of effectiveness was reduction of pain as measured by 24-h NRS pain assessment scale for up to 12 weeks after application of the patch. After the application of the patch, statistically significant reduction of pain in NRS scale was obtained, respectively, after 8 days in the first group with symptoms occurring <648.07 mg/m^2^ of oxaliplatin 3.83 and 2.25 in the second group (*p* = 0.02). After 12 weeks following the application of Qutenza patch, patients achieved a reduction of at least 1.16 in NRS score in the first group compared with 0.25 of the second group (*p* = 0.04). All the results are presented in Table 2.

**Table 2:** Comparison of patients according to dosage after which symptoms occurred

	Initial symptoms after <648.07 mg/m^2^	Initial symptoms after ≥648.07 mg/m^2^	
	*n* = 6	*n* = 12	*p* value
Age, years (SD)	63.17 ± 6.55	62.41 ± 9.24	0.86
Female (%)	5(83.33)	7(58.33)	0.29
Initial pain level	7.67 ± 0.51	7.17 ± 1.34	0.40
Pain after 1 day, NRS	5.50 ± 1.57	4.75 ± 1.36	0.30
Pain after 8 days, NRS	3.83 ± 1.47	2.25 ± 1.14	0.02
Pain after 8 weeks, NRS	2.50 ± 1.87	1.25 ± 1.29	0.11
Pain after 12 weeks, NRS	1.16 ± 1/32	0.25 ± 0.45	0.04
Pain reduction after 1 day, % (SD)	27.98 ± 19.74	33.44 ± 15.20	0.52
Pain reduction after 8 days, % (SD)	49.40 ± 20.34	69.55 ± 12.09	0.02
Pain reduction after 8 weeks, % (SD)	66.67 ± 24.99	84.21 ± 16.20	0.09
Pain reduction after 12 weeks, % (SD)	83.93 ± 18.59	96.96 ± 5.56	0.04

## Discussion

In our study, we described efficacy and safety of high-dose 8% capsaicin patch in treatment of patients with chemotherapy-induced peripheral neuropathy in patients treated with oxaliplatin-based chemotherapy for colon cancer. Total reduction of pain ranged between 84 and 97% assessed at 12 weeks. Furthermore, we observed no adverse events showing that high-dose capsaicin is safe and effective way to treat CIPN.

Effectiveness of high-dose 8% capsaicin is well established in treatment of HIV-associated neuropathy and post-herpetic neuralgia [16]. Results of meta-analysis calculating the data from seven studies incorporating 1458 patients showed reduction of neuropathy-related symptoms in favor of high 8% capsaicin in comparison with low-dose capsaicin 0.04% patch [16]. Another meta-analysis performed by Mou et al. [17] showed that for initial symptoms reduction of >30% occurred in 44% of patients with post-herpetic neuralgia and 41% of patients with HIV-associated neuropathy. All of the mentioned studies have observation period of 2–12 weeks as in our study. In this context, our study shows a striking reduction of neuropathy-related symptoms after application of high-dose 8% capsaicin with symptoms relief up to 97% after 12 weeks.

Certain authors attempted to determine possible predictors of treatment response to treatment with high-dose 8% capsaicin. Katz et al. [18] found that baseline pain intensity score ≤4, absence of allodynia and presence of hypoesthesia are related to sustained response to capsaicin treatment. Mainka et al. [19] found that the presence of hyperalgesia predicts analgesic efficacy of high-dose capsaicin. Our study group unfortunately was too small to calculate possible predictors of treatment response.

Our study has certain limitations. Firstly, our study population was only 18 patients and was limited by the amount of founding for off-label use of patches that we were able to provide. Second limitation that our study group was not examined by standardized questionnaires such as Brief Pain Inventory or McGill Pain inventory. On the other hand, all of patients were examined by a board-certified neurologist. This raw data show much more detailed signs and symptoms specific to neuropathic patients. Most of the standardized questionnaires do not include such specific symptoms. We are confident that in term of presenting neuropathic patients symptoms detailed neurological examinations have deeper significance. Thirdly, we were able to present only observation period of 12 weeks, not being able to determine the total length of effect of the treatment. We are planning to do so in further studies.

To the best of our knowledge this is the first study attempting to determine the use of high-dose 8% capsaicin patch in treatment of chemotherapy-induced peripheral neuropathy.

## References

[1] Windebank A, Grisold W (2008). Chemotherapy-induced neuropathy. J Peripher Nerv Syst.

[2] Mols F, Beijers T, Vreugdenhil G (2014). Chemotherapy-induced peripheral neuropathy and its association with quality of life: a systematic review. Support Care Cancer.

[3] Chu SH, Lee YJ, Lee ES (2015). Current use of drugs affecting the central nervous system for chemotherapy-induced peripheral neuropathy in cancer patients: a systematic review. Support Care Cancer.

[4] Padman S, Lee J, Kumar R (2015). Late effects of oxaliplatin-induced peripheral neuropathy (LEON)–cross-sectional cohort study of patients with colorectal cancer surviving at least 2 years. Support Care Cancer.

[5] Pachman DR, Watson JC, Lustberg MB (2014). Management options for established chemotherapy-induced peripheral neuropathy. Support Care Cancer.

[6] Yang YH, Lin JK, Chen WS (2012). Duloxetine improves oxaliplatin-induced neuropathy in patients with colorectal cancer: an open-label pilot study. Support Care Cancer.

[7] Durand JP, Brezault C, Goldwasser F (2003). Protection against oxaliplatin acute neurosensory toxicity by venlafaxine. Anticancer Drugs.

[8] Rao RD, Michalak JC, Sloan JA (2007). Efficacy of gabapentin in the management of chemotherapy-induced peripheral neuropathy: a phase 3 randomized, double-blind, placebo-controlled, crossover trial (N00C3). Cancer.

[9] Cartoni C, Brunetti GA, Federico V (2012). Controlled-release oxycodone for the treatment of bortezomib-induced neuropathic pain in patients with multiple myeloma. Support Care Cancer.

[10] Fallon MT, Storey DJ, Krishan A (2015). Cancer treatment-related neuropathic pain: proof of concept study with menthol-a TRPM8 agonist. Support Care Cancer.

[11] Gewandter JS, Mohile SG, Heckler CE (2014). A phase III randomized, placebo-controlled study of topical amitriptyline and ketamine for chemotherapy-induced peripheral neuropathy (CIPN): A University of Rochester CCOP study of 462 cancer survivors. Support Care Cancer.

[12] Wagner T, Poole C, Roth-Daniek A (2013). The capsaicin 8% patch for neuropathic pain in clinical practice: a retrospective analysis. Pain Med.

[13] Backonja M, Wallace MS, Blonsky ER (2008). NGX-4010, a highconcentration capsaicin patch, for the treatment of postherpetic neuralgia: a randomised, double-blind study. Lancet Neurol.

[14] Simpson DM, Brown S, Tobias J (2008). Controlled trial of high-concentration capsaicin patch for treatment of painful HIV neuropathy. Neurology.

[15] Derry S, Sven-Rice A, Cole P (2013). Topical capsaicin (high concentration) for chronic neuropathic pain in adults. Cochrane Database Syst Rev.

[16] Mou J, Paillard F, Turnbull B (2013). Efficacy of Qutenza^®^ (capsaicin) 8% patch for neuropathic pain: a meta-analysis of the Qutenza Clinical Trials Database. Pain.

[17] Mou J, Paillard F, Turnbull B (2014). Qutenza (capsaicin) 8% patch onset and duration of response and effects of multiple treatments in neuropathic pain patients. Clin J Pain.

[18] Katz NP, Mou J, Paillard FC (2015). Predictors of response in patients with postherpetic neuralgia and HIV-associated neuropathy treated with the 8% Capsaicin Patch (Qutenza). Clin J Pain.

[19] Mainka T, Malewicz NM, Baron R (2016). Presence of hyperalgesia predicts analgesic efficacy of topically applied capsaicin 8% in patients with peripheral neuropathic pain. Eur J Pain.

